# The association between digital literacy and cognitive function in older adults: the multiple mediating roles of self-efficacy, social participation, and psychological resilience

**DOI:** 10.3389/fragi.2026.1902964

**Published:** 2026-09-04

**Authors:** Rui Ren, Reyisaimu Wumaierjiang, Rui Li, Rui Hou, Kangjia Yang

**Affiliations:** 1 School of Nursing, Wuhan University, Wuhan, China; 2 Department of Nursing, Fengfeng General Hospital of North China Medical & Health Group, HanDan, China; 3 School of Public Health, Shihezi University, Shihezi, China; 4 Key Laboratory for Prevention and Control of Emerging Infectious Disease and Public Health Security, The Xinjiang Production and Construction Corps, Shihezi, China

**Keywords:** cognitive function, digital literacy, psychological resilience, self-efficacy, social participation

## Abstract

**Background:**

Digital literacy refers to the ability to safely and effectively use digital technologies to access, manage, and apply information for daily living and social participation, and has been recognized as a key strategy for promoting healthy aging. However, limited research has simultaneously examined multiple psychosocial mechanisms underlying the relationship between digital literacy and cognitive function in older adults. This study aimed to examine the parallel mediating roles of self-efficacy, social participation, and psychological resilience in the relationship between digital literacy and cognitive function.

**Methods:**

A cross-sectional design was employed. We recruited older adults aged ≥60 years from a city in China to complete a questionnaire survey. The survey instruments included a general information questionnaire, the Digital Literacy Scale for Older Adults, the General Self-Efficacy Scale, the Social Participation Scale for Older Adults, the Brief Psychological Resilience Scale, and the Mini-Mental State Examination (MMSE). A multiple mediation model was constructed using structural equation modeling (SEM).

**Results:**

Among the 630 participants, the largest proportion of participants were aged 70–79 years (45.9%), and 52.1% were male. The mean MMSE score was 25.71 (SD = 3.38), indicating that the sample, on average, fell within the normal cognitive range (scores below 24 are typically considered indicative of mild cognitive impairment). Digital literacy was significantly correlated with cognitive function (*r* = 0.246, *P* < 0.01). The total effect of digital literacy on cognitive function was 0.347 (95% CI 0.258–0.417, *P* = 0.002). Mediation analysis revealed that digital literacy indirectly influenced cognitive function through three pathways: self-efficacy, social participation, and psychological resilience. The total indirect effect accounted for 39.5% of the total effect, and the direct effect accounted for 60.8%. Among the three mediating pathways, self-efficacy showed the strongest indirect effect (16.4%), followed by social participation (13.0%) and psychological resilience (10.1%).

**Conclusion:**

Digital literacy not only shows a direct association with cognitive function in older adults but also shows indirect associations through self-efficacy, social participation, and psychological resilience. Therefore, multifaceted, synergistic interventions may be considered that integrate digital skills training with social engagement and psychological support are associated with cognitive health and the quality of life for older adults.

## 1 Introduction

Against the backdrop of accelerating global population aging, China is facing a particularly severe aging situation. It is projected that by 2050, the proportion of older adults aged 60 years and above in China will reach 26.1%, with the total population reaching 366 million (Feng et al., 2020; United Nations, 2019). With the rapid growth of the older adult population, cognitive health has increasingly become a focal point in public health. Cognitive function, as a core indicator of higher-order executive brain activities, is essential for maintaining independence and quality of life in later adulthood. During aging, the brain undergoes biological changes, including white matter reduction, prefrontal cortex volume loss, and declines in neurotransmitter levels, that are associated with age-related cognitive changes (Gonzales et al., 2022; Juraska and Chisholm, 2011). Additionally, aging-related vascular dysfunction may be associated with an increased risk of chronic and neurodegenerative diseases (Wright et al., 2011; Barnes, 2015). These biological vulnerabilities underscore why preserving cognitive function is critical for healthy aging. Notably, cognitive decline serves as a strong predictor of disability and loss of independence in daily living among older adult (Xue et al., 2022), and cognitive impairment is often associated with reduced self-care capacity and increased reliance on caregivers (Thoma-Lürken et al., 2018). Beyond these functional consequences, cognitive decline is also intricately linked with psychological wellbeing, as persistent psychological distress has been shown to be strongly associated with subsequent dementia (Stafford et al., 2026). In China, the substantial population at preclinical stages of cognitive decline further underscores the urgency of identifying modifiable protective factors. Importantly, the 2020 Lancet Commission on dementia prevention, intervention, and care reported that approximately 40% of dementia cases are attributable to a range of modifiable risk factors across the life course (Livingston et al., 2020). This finding provides an important theoretical basis for developing behavioral interventions that may be associated with cognitive health maintenance. Nevertheless, most patients have insufficient awareness of the condition and fail to recognize its potential risk of progression (Jia et al., 2020). Furthermore, current clinical practice still lacks pharmacological therapies that can effectively reverse cognitive decline (Karakaya et al., 2013), underscoring the importance of non-pharmacological intervention approaches. Against this background, actively exploring modifiable psychosocial and lifestyle factors has become a key strategic direction for identifying factors that may be associated with cognitive health maintenance and healthy aging.

According to the 56th Statistical Report released by the China Internet Network Information Center (CNNIC), as of June 2025, the number of older adult internet users aged 60 years and above in China reached 161 million, with an internet penetration rate of 52.0%. Based on this estimate, approximately 149 million older adults remain unconnected to the internet (Center, 2025). Against the backdrop of the accelerating digitalization of the older adult population, digital literacy is considered a key foundation for individuals to effectively integrate into the digital world. As defined by the United Nations Educational, Scientific and Cultural Organization (UNESCO), digital literacy refers to a comprehensive set of abilities that enable individuals to access, understand, analyze, evaluate, and creatively utilize information using digital devices and technologies, as well as to effectively employ digital tools in work and daily life, and to actively participate in socio-economic and cultural activities (Law et al., 2018). In recent years, researchers have recognized the value of digital literacy in geriatric health. Existing studies have shown that digital literacy is closely associated with health information acquisition (Meng, 2025), improvement in quality of life (Xin et al., 2025), and changes in attitudes toward aging (Di and Wang, 2025) among older adults. However, when examining the relationship between digital literacy and cognitive function in older adults, the existing literature often exhibits a specific limitation in research perspective. Most studies have treated cognitive function merely as a background variable or confounding factor, with primary attention focused on beneficial associations of internet use with cognitive function (Duan et al., 2024; Jin et al., 2019), thereby overlooking the intrinsic association and specific pathways between digital literacy and cognitive function. Systematically exploring the pathways through which digital literacy may be related to cognitive function in older adults would not only help identify factors associated with cognitive health in the digital aging era but also provide a theoretical basis for developing targeted prevention or health promotion strategies.

Conservation of Resources (COR) theory offers an important perspective on this mechanism. The theory posits that individuals may adopt resource investment to obtain valuable returns and offset losses (Hobfoll, 1989; Hobfoll et al., 1990). It further emphasizes that acquiring one key resource facilitates the acquisition and mobilization of others, generating a gain spiral that expands resource reservoirs and fosters further growth. This mechanism is central to enhancing individuals’ capacity to withstand and recover from stress (Hobfoll, 2001; Hobfoll, 2002). In the context of digital aging, digital literacy can be conceptualized as a novel personal resource that may facilitate further resource gains through resource investment, whereas cognitive function represents a foundational resource for independent living and successful aging. For older adults, social participation and digital technology use constitute important gainful resources that replenish resource reservoirs and buffer against age-related losses and stress (Roquet et al., 2024). As personal resources tend to decline with age, rendering older adults vulnerable to social isolation and loneliness (Hawkins-Elder et al., 2018), COR theory suggests that older adults may adopt compensatory strategies to counteract resource loss. Digital literacy, as an internalized capability, can drive the mobilization of other resources.

**FIGURE 1:**
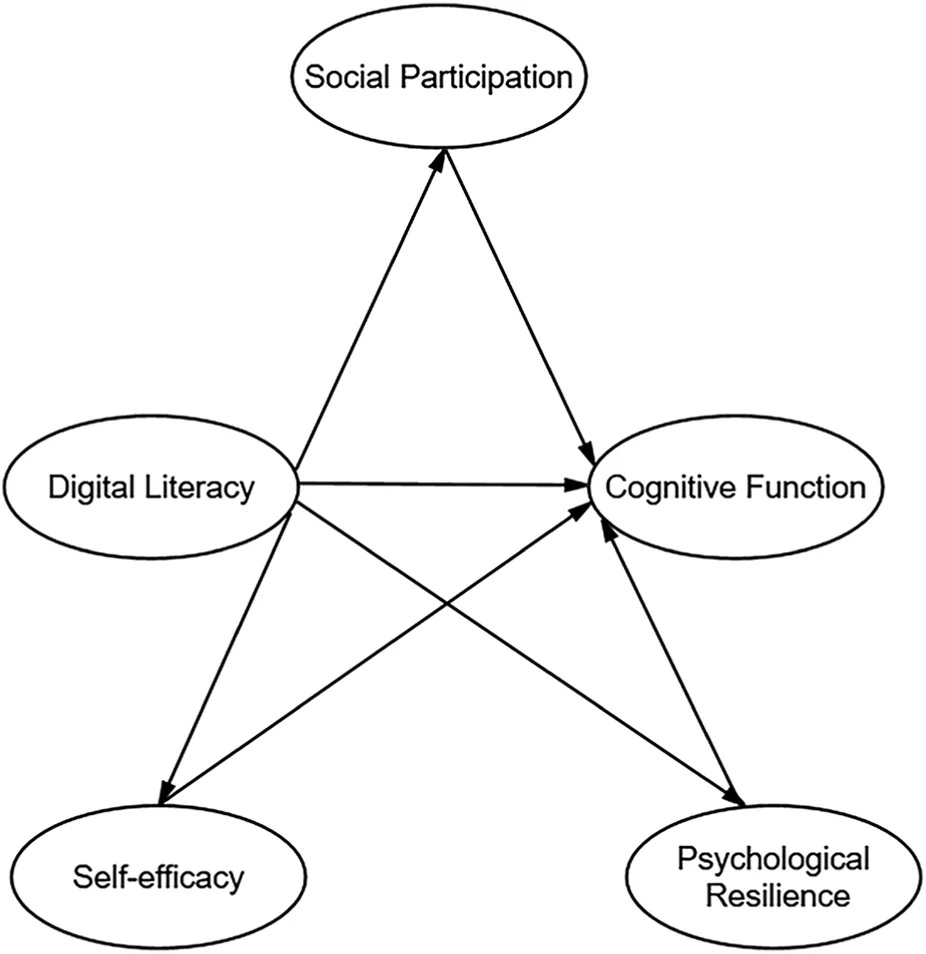
A parallel multiple mediation hypothesis model of the relationship between digital literacy and cognitive function.

Drawing on this theoretical perspective, we propose that digital literacy may be associated with cognitive function through three distinct yet interrelated pathways: the intrapersonal pathway of self-efficacy, the interpersonal pathway of social participation, and the adaptive pathway of psychological resilience. At the intrapersonal level, self-efficacy is an important intrinsic quality that reflects an individual’s level of confidence in their ability to accomplish specific tasks (Bandura, 1978). Digital literacy is associated with higher self-efficacy, which promotes proactive health behaviors and cognitive engagement that may be associated with slower cognitive decline (Rimmele et al., 2022; Assunção and Chariglione, 2023; Di and Wang, 2025). At the interpersonal level, digital literacy is associated with social participation, which provides cognitive stimulation that may contribute to cognitive maintenance (Cohn-Schwartz et al., 2021; Evans et al., 2019; Hou et al., 2024; Stern, 2012). At the adaptive level, digital literacy is associated with psychological resilience, which helps older adults cope with age-related challenges and preserve resources (De Lucia et al., 2025; Jung et al., 2021; Hobfoll et al., 2018). Digital technology is also associated with social interaction, thereby reducing social isolation and stress (Hobfoll, 2002), and resilient individuals are better able to protect existing resources and resist resource loss spirals (Hobfoll, 2001). Collectively, these three pathways operate via resource investment and gain spiral mechanisms. Accordingly, this study examines these three variables as parallel mediators, testing the applicability of COR theory in the context of digital aging. In summary, digital literacy, self-efficacy, social participation, psychological resilience, and cognitive function are closely linked in older adults. However, most studies have examined these relationships only in pairs and in isolation. An integrated model is still lacking to reveal how self-efficacy, social participation, and psychological resilience jointly mediate the association between digital literacy and cognitive function. Based on Conservation of Resources theory, this study builds a framework to systematically examine these mediating pathways in older adults (Figure 1).

We set out to test the following hypotheses:

H1 Digital literacy has a significant positively associated with cognitive function in older adults.

H2 Self-efficacy mediates the association between digital literacy and cognitive function in older adults.

H3 Social participation mediates the association between digital literacy and cognitive function in older adults.

H4 Psychological resilience mediates the association between digital literacy and cognitive function in older adults.

## 2 Methods

### 2.1 Study design, setting, and participants

This cross-sectional study was conducted in Handan, Hebei, China, from December 2025 to February 2026. A pilot test (n = 30) was conducted in December 2025 to assess feasibility. Formal ethics approval was obtained, and data were collected from January to February 2026. Convenience sampling was used to recruit older adults aged 60 years and above from a tertiary hospital, as well as surrounding township hospitals and communities. Inclusion criteria were: (1) age ≥60 years; (2) ability to communicate effectively; and (3) prior experience using digital devices (e.g., smartphones, tablet computers). Exclusion criteria were: (1) MMSE score ≤20 (indicating moderate-to-severe cognitive impairment) (Perneczky et al., 2006); (2) diagnosed mental illness; or (3) physical disability preventing completion of the questionnaire, including severe visual impairment (e.g., inability to read text on a smartphone screen), severe hearing impairment (e.g., inability to understand verbal instructions even with hearing aids), or any other condition that would prevent the participant from independently understanding and responding to the questionnaire items, even with research-staff assistance. All voluntarily participating older adults provided informed consent. Investigators received certain professional training prior to data collection.

The sample size was determined based on Kendall’s rule of thumb, which recommends a sample size of 5–10 times the number of independent variables (Chen, 2012). A total of 33 variables were included in this study: 15 demographic items, 5 dimensions of digital literacy, 1 dimension of self-efficacy, 6 dimensions of social participation, 1 dimension of psychological resilience, and 5 dimensions of the MMSE. The required sample size was calculated as 33 multiplied by 10, yielding a target of 330 participants. Accounting for a potential 20% rate of invalid questionnaires, the required sample size was at least 413 participants. To further increase statistical power, meet the requirements of structural equation modeling (Kline, 2023), and ensure the stability of the multiple mediation model estimates, a total of 630 older adults were ultimately enrolled in this study.

Participants completed the online survey through the Questionnaire Star platform during face-to-face interviews, with investigators inputting responses into the platform. The MMSE was uniformly administered by trained interviewers to all participants through verbal questioning, where the Questionnaire Star platform served solely as an online tool. Participants who could read screen information independently completed the remaining self-report questionnaires by themselves. The study protocol was approved by the Ethics Committee of Fengfeng General Hospital of North China Medical and Health Group (Approval No: 2026B10), and all study procedures were conducted in accordance with the ethical guidelines of the Declaration of Helsinki. All participants provided written informed consent.

### 2.2 Data collection

#### 2.2.1 Demographic characteristics

A self-designed questionnaire was used to collect demographic information, consisting of 15 items, including age, gender, marital status, educational level, and other basic demographic characteristics.

#### 2.2.2 Digital literacy scale for older adults

Digital literacy was measured using the scale developed by Dai and Luo (2024). The scale consists of 15 items across five dimensions: information acquisition, communication, content creation, safety awareness, and problem solving. Each item is rated on a five-point Likert scale (1 = “strongly disagree” to 5 = “strongly agree”), with total scores ranging from 15 to 75. This study adopted the total score to reflect overall digital literacy. Higher scores indicate greater digital literacy. The Cronbach α coefficient was 0.94 for the original scale and 0.978 for the present sample.

#### 2.2.3 General self-efficacy scale (GSES)

The Chinese version of the General Self-Efficacy Scale was adopted in this study, which was originally developed by Schwenzer and colleagues (Zhang and Schwarzer, 1995) and translated and validated among Chinese populations by Huang Fei and colleagues (Huang F, 2010). The scale consists of 10 items, each rated on a 4-point Likert scale ranging from 1 (“completely incorrect”) to 4 (“completely correct”). Total scores range from 10 to 40, with higher scores indicating greater general self-efficacy. The Cronbach α coefficient of the original scale was 0.96, and in the present study, it was 0.953.

#### 2.2.4 Social participation scale for older adults

This study employed the Social Participation Scale for Older Adults developed by Ren et al. (2024). The scale consists of 22 items covering six dimensions: household activities, leisure activities, visits with relatives and friends, group recreational activities, volunteer and public welfare activities, and online participation. All items were rated on a five-point Likert scale (1 = “completely inconsistent” to 5 = “completely consistent”), with total scores ranging from 22 to 110. Higher scores indicate higher levels of social participation. The Cronbach α coefficient of the original scale was 0.86, and the Cronbach α coefficient in the present study was 0.979.

#### 2.2.5 Brief psychological resilience scale (CD-RISC-10)

Psychological resilience was measured using the 10-item Connor-Davidson Resilience Scale (CD-RISC-10), originally developed by Campbell-Sills and colleagues (Hoyl et al., 1999) and subsequently translated into Chinese by Zhang and Li (2018). The scale consists of 10 items, each rated on a five-point Likert scale (0 = “never” to 4 = “always”). Total scores range from 0 to 40, with higher scores indicating greater psychological resilience. The Cronbach α coefficient was 0.85 for the original scale and 0.948 for the present study.

#### 2.2.6 Mini-mental state examination (MMSE)

The MMSE was originally developed by Folstein et al. (1975) in the United States (Folstein et al., 1975), and the Chinese version translated and revised by Zhou Xiaoxuan (XZ, 2015), was used to assess cognitive function. The scale consists of 30 items covering cognitive domains including orientation, registration, attention and calculation, recall, and language. Each item is scored one point, with total scores ranging from 0 to 30. Higher scores indicate better cognitive function. The Cronbach α coefficient of the original scale was 0.833, and the Cronbach α coefficient in the present study was 0.701.

### 2.3 Statistical analysis

Data analysis and model construction were conducted via SPSS 25.0 and AMOS 28.0. Cronbach α coefficient was utilized to evaluate scale reliability. Continuous variables were described as mean (SD), while categorical variables were expressed as frequencies (percentages). Pearson correlation analysis was adopted to explore bivariate relationships. Multiple linear regression was conducted to explore the predictive associations between study variables. Based on previous studies (Baumgart et al., 2015; Livingston et al., 2020; LaPlume et al., 2022), the following covariates were adjusted for in the analyses: age, education level, smoking status, hearing problems, and hypertension. Structural equation modeling was employed to test the research hypotheses via a multiple mediation model. Model goodness-of-fit was assessed using the following indices: *χ* ^*2*^/df, CFI, TLI, and RMSEA. Mediating effects were tested using the bias-corrected bootstrap method with 5,000 resamples to compute 95% confidence intervals. The significance level was set at *P* < 0.05 for all analyses.

## 3 Results

### 3.1 Participant characteristics

A total of 630 older adults were included in this study (Table 1). With respect to sociodemographic characteristics, the largest age group was 70–79 years (45.9%). The sex distribution was relatively balanced (52.1% male). Regarding marital status, 80.3% were married, of whom 57.3% lived with their spouse. In terms of educational level, junior high school (30.8%) and primary school (28.7%) were the most common categories. Monthly personal income was predominantly concentrated in the ranges of 2000–3000 yuan (29.2%) and 3000–5000 yuan (29.5%). With respect to health and behavioral characteristics, non-smokers accounted for 70.6%, and alcohol consumers accounted for 41.7%. Regarding sleep duration, 49.4% slept 6–8 h per day, while 33.2% slept less than 6 h. Older adults who used the internet for 1–2 h per day accounted for 43%. In terms of health conditions, the three most prevalent chronic diseases were hypertension (30.6%), diabetes mellitus (17.6%), and hyperlipidemia (16.8%). The primary means of acquiring digital skills were self-learning (78.3%) and learning from their children (69%).

**TABLE 1:** Participant characteristics of the study sample (N = 630).

Variable	Category	n	%
Sociodemographic characteristics			
Age	60–69	262	41.6
	70–79	289	45.9
	80–89	79	12.5
Sex	Male	328	52.1
	Female	302	47.9
Marital status	Single	2	0.3
	Married	506	80.3
	Widowed	81	12.9
	Divorced	41	6.5
Educational level	No formal education	22	3.5
	Primary school	181	28.7
	Middle school	194	30.8
	High school/Secondary vocational	157	24.9
	Associate degree	45	7.1
	Bachelor’s degree	28	4.4
	Graduate degree	3	0.5
Residential status	Living alone	85	13.5
	Living with spouse	361	57.3
	Living with children	148	23.5
	Living with others	36	5.7
Residential type	Urban	373	59.2
	Rural	257	40.8
Personal income	<1000	49	7.8
	1000–2000	81	12.9
	2000–3000	184	29.2
	3000–5000	186	29.5
	5000–8000	94	14.9
	8000–12000	26	4.1
	>12,000	10	1.6
Household income	<2000	40	6.3
	2000–5000	197	31.3
	5000–10000	217	34.4
	10,000–20000	136	21.6
	>20,000	40	6.3
Health and behavioral characteristics			
Smoking	No	445	70.6
	Yes	185	29.4
Alcohol consumption	No	367	58.3
	Yes	263	41.7
Chronic disease	No	332	52.7
	Hypertension	193	30.6
	Diabetes	111	17.6
	Heart disease	81	12.9
	Hyperlipidemia	106	16.8
	Cerebrovascular disease	38	6.0
	Neurological disease	45	7.1
	Cancer	9	1.4
	Other	5	0.8
Sleep	6–8 h/day	311	49.4
	<6 h/day	209	33.2
	≥8 h/day	110	17.5
Hearing problems	No	460	73
	Yes	170	27
Daily online time	<1 h	176	27.9
	1–2 h	271	43
	>2 h	183	29
Digital skills	Self-learning	493	78.3
	Taught by children	435	69
	Community	293	46.5
	Friends	184	29.2
	AI tools	43	6.8

multiple responses allowed for chronic disease and digital skills; percentages may exceed 100%.

### 3.2 Descriptive statistics and bivariate correlations

The means, standard deviations, and Pearson correlation coefficients among study variables are presented in Table 2. The mean digital literacy score was 52.84 (SD = 14.38), and the mean MMSE cognitive function score was 25.71 (SD = 3.38). All variables were significantly positively correlated with each other (*P* < 0.01). Digital literacy showed moderate positive correlations with self-efficacy (*r* = 0.441) and social participation (*r* = 0.406), and weak positive correlations with psychological resilience (*r* = 0.254) and cognitive function (*r* = 0.246). Self-efficacy was relatively strongly correlated with social participation (*r* = 0.460) and psychological resilience (*r* = 0.426). Cognitive function was weakly correlations with digital literacy (*r* = 0.246), self-efficacy (*r* = 0.242), social participation (*r* = 0.228), and psychological resilience (*r* = 0.185).

**TABLE 2:** Means, standard deviations, and correlations among study variables.

Variable	Digital literacy	Self-efficacy	Social participation	Psychological resilience	Cognitive function
Digital literacy	**1.000**	-	-	-	-
Self-efficacy	0.441**	**1.000**	-	-	-
Social participation	0.406**	0.460**	**1.000**	-	-
Psychological resilience	0.254**	0.409**	0.350**	**1.000**	-
Cognitive function	0.246**	0.242**	0.228**	0.185**	**1.000**
M	52.84	32.74	78.77	25.87	25.71
SD	14.38	7.65	20.27	8.88	3.38

M = mean; SD, standard deviation. Bold values (1.000) denote the correlation of each variable with itself. All other variables (self-efficacy, social participation, psychological resilience, and cognitive function) are presented as total scores. **P < 0.01.

### 3.3 Multiple linear regression analysis

Multiple linear regression analyses were conducted to examine the associations between digital literacy and cognitive function, as well as the proposed mediating variables. As shown in Table 3, after controlling for well-established demographic confounders, including age, education level, smoking, hearing problems, and hypertension (Livingston et al., 2020; Baumgart et al., 2015; LaPlume et al., 2022). Digital literacy remained significantly and positively associated with cognitive function, consistent with the unadjusted model. Specifically, digital literacy was significantly and positively associated with cognitive function (standardised *β* = 0.247, *P* < 0.001), accounting for 5.9% of the total variance. Furthermore, digital literacy was significantly and positively associated with self-efficacy (*β* = 0.168, *P* < 0.001), social participation (*β* = 0.154, *P* < 0.001), and psychological resilience (*β* = 0.133, *P* = 0.001), explaining 8.0%, 7.7%, and 7.4% of the variance in each outcome, respectively. The variance inflation factor (VIF) values for all models were below 2, indicating no serious multicollinearity among the variables.

**TABLE 3:** Multiple regression analysis of digital literacy on cognitive function and mediating variables.

Model	Variables	B	*β*	*t*	*R2*	*P*	VIF
Model 1	Digital literacy	0.058	0.247	6.380	0.059	<0.001	1.001
Model 2	Digital literacy - self-efficacy	0.074	0.168	3.932	0.080	<0.001	1.244
	Digital literacy - social participation	0.026	0.154	3.668	0.077	<0.001	1.198
	Digital literacy - psychological resilience	0.050	0.133	3.329	0.074	0.001	1.077

*β* = standardized regression coefficient; VIF, variance inflation factor; *R* ^*2*^ = adjusted *R* ^*2*^. All models adjusted for covariates (age, education, income, smoking, hypertension, hearing problems; all P > 0.05). Dependent variable: cognitive function. Model 1: + Digital Literacy; Model 2: + Digital Literacy + one mediator (self-efficacy, social participation, or psychological resilience; all P < 0.05).

### 3.4 Mediation analysis

**FIGURE 2:**
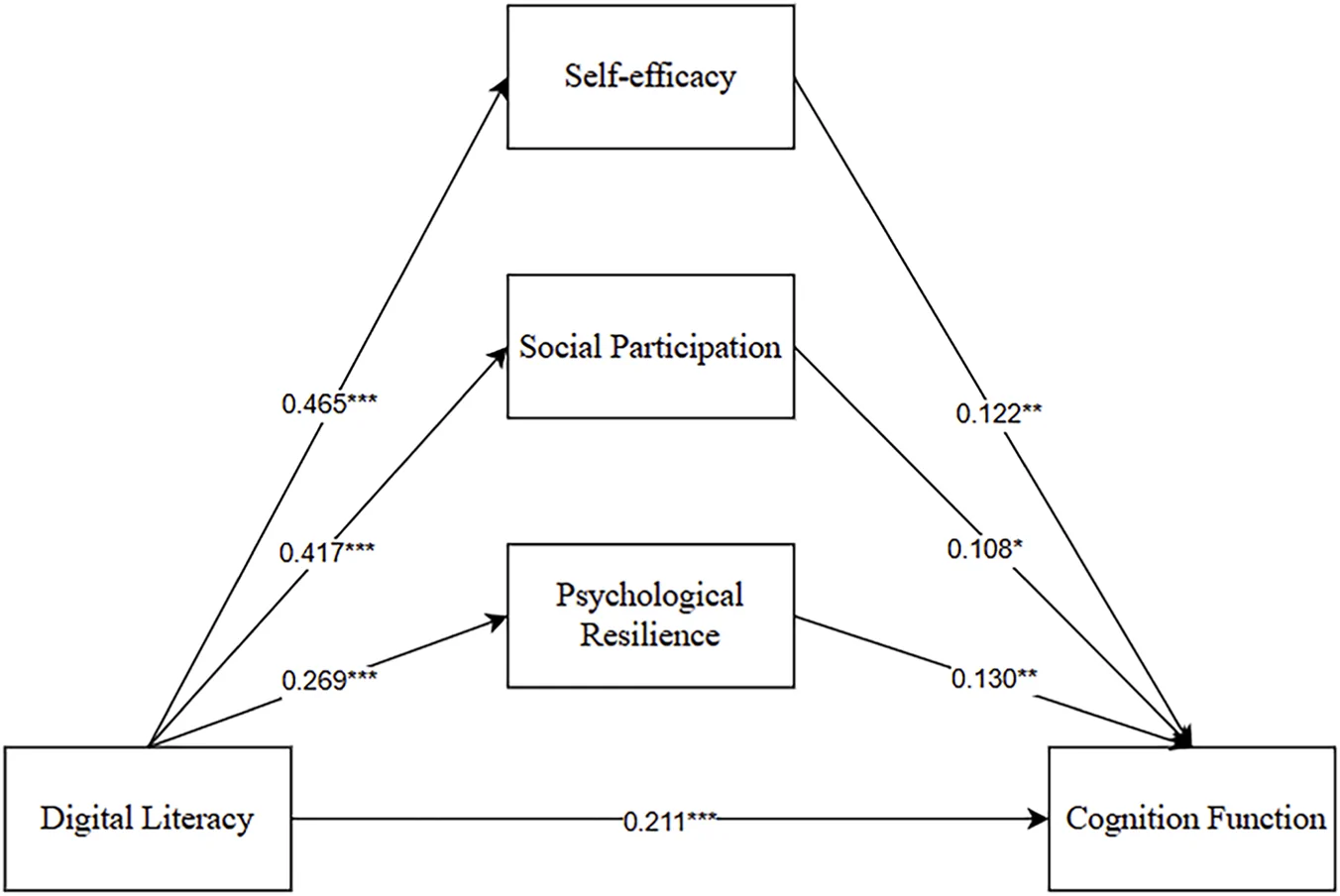
Multiple mediation model linking digital literacy to cognitive function. Standardized path coefficients are presented, based on 5,000 bootstrap samples.

SEM was employed to examine the multiple mediating roles of self-efficacy, social participation, and psychological resilience in the relationship between digital literacy and cognitive function in older adults. The results indicated a good model fit: *χ* ^*2*^/df = 3.253, RMSEA = 0.060 (90% CI 0.057–0.063), CFI = 0.944, TLI = 0.940, with all fit indices falling within acceptable ranges. Figure 2 and Table 4 present the structural equation model of the mediating pathways and the effect decomposition results. The results showed that the total association between digital literacy and cognitive function in older adults was significant (*β* = 0.347,95% CI 0.258–0.417, *P* = 0.002). After controlling for the mediating variables, the direct statistical association between digital literacy and cognitive function remained significant, suggestingan independent statistical association between digital literacy and cognitive function. The hypothesis testing results are summarized as follows:

**TABLE 4:** Decomposition of direct, indirect, and total effects of digital literacy on cognitive function via mediators.

Effect type	Path	*β*	Boot SE	95% CI	Effect ratio (%)	*P*
Indirect effect	DL → SE → CF	0.057	0.026	0.006–0.109	16.4	0.026
	DL → SP → CF	0.045	0.023	0.000–0.090	13.0	0.047
	DL → PR → CF	0.035	0.014	0.008–0.065	10.1	0.013
Direct effect	DL → CF	0.211	0.050	0.106–0.302	60.8	0.001
Total effect	​	0.347	0.039	0.258–0.417	100	0.002

Abbreviations: DL, digital literacy; SE, Self-efficacy; SP, social participation; PR, psychological resilience; CF, cognitive function; Boot SE, bootstrap standard error; CI, confidence interval. All path coefficients are significant at *P* < 0.05 (two-tailed). Bias-corrected bootstrap with 5,000 resamples was used to compute 95% CIs, for indirect effects.

H5 Digital literacy showed a significant direct positive statistical association with cognitive function in older adults (*β* = 0.211, 95% CI 0.106–0.302, *P* = 0.001), accounting for 60.8% of the total effect.

H6 The indirect pathway through self-efficacy was significant (*β* = 0.057, 95% CI 0.006–0.109, *P* = 0.026), explaining 16.4% of the total effect.

H7 The indirect pathway through social participation showed the association (*β* = 0.045, 95% CI 0.000–0.090, *P* = 0.047), accounting for 13% of the total effect.

H8 Although the indirect effect of psychological resilience was relatively small, it remained statistically significant (*β* = 0.035, 95% CI 0.008–0.065, *P* = 0.013), accounting for 10.1% of the total effect.

The total indirect effect through the three mediating pathways was significant, accounting for 39.5% of the total statistical association between digital literacy and cognitive function. These findings suggest that self-efficacy, social participation, and psychological resilience mediate the relationship between digital literacy and cognitive function.

## 4 Discussion

This study found a significant association between digital literacy and cognitive function in older adults, mediated by self-efficacy, social participation, and psychological resilience. The mean digital literacy score indicated a moderate level (score rate: 70.5%), which is consistent with the findings of Shin and colleagues (Shin et al., 2025), who used the same Digital Competence Framework (DigComp)-based framework. The mean MMSE score in our sample was 25.71, higher than the 25.3 reported in a comparable study (Hong et al., 2023) but lower than the median 29 in the DO-HEALTH study (Kistler-Fischbacher et al., 2025). The main reasons for this difference are the lower educational level of our sample (mostly primary and junior high school) and the inclusion of hospital-based sub-healthy individuals, resulting in greater sample heterogeneity. Digital literacy showed a direct statistical association with cognitive function. More importantly, it also showed indirect statistical associations through three psychosocial pathways: self-efficacy, social participation, and psychological resilience. This finding differs from prior studies that typically focused on a single mediating pathway (Di and Wang, 2025; Lin et al., 2023); the present study incorporated self-efficacy, social participation, and psychological resilience into a unified model, simultaneously testing the relative contributions of all three pathways. These findings provide a theoretical foundation for understanding how digital resources may be associated with cognitive function in older adults through the mobilization of psychological and social resources, and they hold important practical value for both cognitive health maintenance and digital inclusion in the aging population.

### 4.1 Direct effect of digital literacy on cognitive function in older adults

The direct association between digital literacy and cognitive function remained positively significant even after accounting for the mediating pathways, suggesting that digital literacy is independently associated with cognitive function in older adults. This finding is consistent with the cognitive reserve hypothesis, which posits that digital technology use is associated with cognitive reserve by providing cognitively complex activities such as learning new skills, processing multi-source information, and solving problems (Benge and Scullin, 2020; Benge and Scullin, 2025). Specifically, technology use often involves complex cognitive operations, including attentional switching, working memory updating, problem-solving, and information retrieval, which are associated with prefrontal lobe function and associated neural networks. From a daily-life perspective, older adults with higher digital literacy are regularly exposed to novel, diverse, and moderately challenging information and activities, which provides continuous stimulation and exercise for the brain, thereby may be associated with slower cognitive decline. In contrast, older adults with lower digital literacy tend to be exposed to relatively monotonous information and activities, making them more susceptible to reduced social interaction and information isolation, which may conversely may be associated with faster cognitive deterioration. This contrast further supports the statistical association between digital literacy in the cognitive aging process. This finding is consistent with previous research (Lee et al., 2022), suggesting that programs targeting digital literacy may be associated with cognitive health in older adults. Future research and practice may consider integrating digital literacy training into basic community-based elderly care services. Governments could foster a multi-stakeholder digital education network involving society, communities, and families. Based on older adults’ specific needs, this network could systematically guide their digital engagement across three dimensions: awareness of digital participation, forms of participation, and pathways to participation. Leveraging the age-friendly renovation projects outlined in the 15th Five-Year Plan, efforts should prioritize developing training curricula focused on social interaction scenarios may be a promising direction. Furthermore, full use of “Silver Age Health Stations” resources may help construct a sustainable pathway for improving digital literacy among older populations.

### 4.2 The mediating role of self-efficacy

This study confirmed that self-efficacy played a significant mediating role in the relationship between digital literacy and cognitive function. As noted in previous studies (Lee et al., 2022; Xin et al., 2025), this finding shows that self-efficacy is a stable psychological pathway through which digital literacy may be associated with the health and wellbeing of older adults. In support of this, according to Bandura’s social cognitive theory (Bandura, 1978), successful experiences are the core source of self-efficacy. Consequently, older adults with higher digital literacy are more likely to obtain positive feedback during technology use, which may strengthen beliefs in their own capabilities and extends the association to cognitive health. At the mechanistic level, neuroimaging evidence supports the association between self-efficacy and cognitive function, showing that self-efficacy has identifiable neural structural correlates. Its level is closely associated with brain structural integrity, which, in turn, is associated with cognitive function (Davis et al., 2012; Nakagawa et al., 2017). Based on these findings, researchers have suggested that self-efficacy may be a modifiable factor for healthy aging (Davis et al., 2012). However, a study by Hu and colleagues (Hu et al., 2026) on intergenerational mentoring found that self-efficacy did not mediate the relationship between digital reverse mentoring and digital literacy in older adults. This contradictory finding reveals a key condition for the effectiveness of self-efficacy. In intergenerational mentoring contexts, older adults’ learning process is predominantly guided by receiving instruction from others, with limited opportunities for independent exploration and problem-solving. Consequently, they struggle to develop the “enactive mastery experiences” emphasized by Bandura and tend to attribute learning outcomes to external help rather than their own efforts, thereby weakening the substantive enhancement of self-efficacy. By contrast, the digital literacy examined in this study reflects older adults’ ability to use digital devices independently. This ability is accumulated through active practice and is therefore more likely to be associated with the development of self-efficacy. It follows that the mediating effect of self-efficacy relies on active engagement during learning, rather than passive acceptance of guidance from others. Accordingly, the design of digital intervention programs for older adults is recommended to emphasize opportunities for independent exploration and problem-solving, thereby fully leveraging the mediating role of self-efficacy in the relationship between digital literacy and cognitive health. Specifically, digital activities organized by community institutions could actively guide and encourage older adults to engage through self-expression, mutual communication, and autonomous participation. Such approaches may be associated with improved emotional wellbeing and higher self-efficacy in older adults.

### 4.3 The mediating role of social participation

Among the three psychosocial pathways, social participation exhibited the indirect effect. This finding suggests that digital literacy may be associated with cognitive function in older adults through its association with social participation. Our findings are consistent with previous evidence demonstrating that digital information and communication systems are associated with social participation in communities, which in turn may be associated with physical and mental health as well as cognitive function in older adults (Li et al., 2025; Lin et al., 2023; Hou et al., 2022; Welch et al., 2016). However, a study by Shiratsuchi and colleagues (Shiratsuchi et al., 2025) found no significant association between volunteer-based social participation and cognitive function, suggesting that merely increasing the frequency or quantity of social participation may be insufficient to support cognitive function; rather, the degree of psychological engagement during participation may be the key factor. This finding does not negate the value of social participation but rather reveals a more nuanced issue: different qualities of social participation may show differential associations with cognitive function. Against this backdrop, the unique role of digital literacy becomes particularly important, as digital technology is associated with older adults’ engagement in more proactive, autonomous social interactions rather than being limited to passive information reception. At the mechanistic level, social participation may be associated with cognitive function through multiple pathways (Kennedy et al., 2017): at the neural level, through associations with neuroplasticity that may support brain function; at the psychophysical level, by improving overall health status to provide fundamental support; and at the social level, by expanding interpersonal networks to enrich the sources of cognitive stimulation. Based on these findings, future digital literacy training programs for older adults may consider incorporating practice modules specifically designed for social participation, helping older adults translate digital skills into tangible social connections. Additionally, expanding the coverage of digital technology service facilities and optimising community infrastructure to provide accessible and convenient support for older adults’ digital engagement, and encouraging older adults to participate in diverse digital activities, including digital consumption, healthcare, and entertainment.

### 4.4 The mediating role of psychological resilience

This study found that psychological resilience mediated the relationship between digital literacy and cognitive function. Previous literature supports both segments of this pathway (Chen et al., 2023; Jung et al., 2021; Nakagawa et al., 2017). Diminished stress-coping ability may indicate early accumulation of tau pathology. Stress may promote the build-up of tau and amyloid proteins during aging; thus, the stress-buffering ability of psychological resilience may be associated with the preventiont or slowing of cognitive decline, suggesting that early intervention may be beneficial (Arenaza-Urquijo et al., 2020). However, Awais Aftab’s study demonstrated that resilience was significantly associated with positive psychological traits such as optimism and social support, but not with cognitive function (Aftab et al., 2022). This discrepancy may be attributable to the type of resilience measured. Aftab and colleagues assessed trait-like resilience across general contexts, which may show only an indirect association with cognitive function. In contrast, the present study focused on practice-based resilience accumulated through digital technology use, which may more directly protect cognitive function. From a neuroimaging perspective (Rooij et al., 2023), the brain activation patterns underlying psychological resilience are context-dependent. When older adults experience successful digital engagement, brain regions responsible for rational thinking and decision-making are activated, which may be associated with higher psychological resilience. Conversely, under stressful conditions, regions associated with mind-wandering and worry are activated, which may be associated with lower resilience. This finding suggests that the association between psychological resilience and cognitive function may depend on specific contexts of activation. In the present study, active technology engagement fostered by digital literacy serves as a key prerequisite for linking psychological resilience to cognitive function. Based on the above analysis, future research should distinguish between the differential effects of trait resilience and practice-oriented resilience and adopt longitudinal designs to examine the longitudinal association of digital literacy interventions on resilience and cognitive function. Specifically, interventions could target the difficulties older adults encounter in acquiring digital skills by providing interpretative guidance, supporting hands-on autonomous practice, and offering psychological counseling and emotional support. These approaches may be associated with improvements in older adults’ mental health and higher their psychological resilience.

## 5 Limitations

Several limitations of this study should be acknowledged. First, face-to-face interviews may have introduced interviewer bias. Second, the inclusion criteria requiring prior experience with digital devices and excluding individuals with MMSE scores below 20 may have introduced selection bias. While this criterion was implemented to ensure participants’ ability to complete the digital literacy assessment, it likely resulted in a sample with relatively higher digital literacy and cognitive functioning, thereby underestimating the proportion of older adults with the lowest levels of digital literacy and the most severe cognitive impairment. Consequently, the generalizability of our findings to the broader population of older adults, particularly to those who are digitally inexperienced or have more advanced cognitive decline, may be constrained. Third, the cross-sectional design precludes any causal inferences regarding the observed relationships. Fourth, the use of self-report measures may have been subject to social desirability bias. Fifth, the sample was drawn solely from community and hospital settings in a single city, which limits the generalizability of the findings. Sixth, this study focused exclusively on psychosocial mechanisms and did not incorporate neurobiological pathways. Additionally, despite adjusting for multiple established covariates, the potential for unmeasured confounding, such as depression, cannot be entirely ruled out. Future research should employ longitudinal designs, objective assessments, and multimodal techniques to further validate the findings. Despite these limitations, this study systematically elucidates the multiple mechanisms through which digital literacy may be associated with cognitive function in older adults, thereby providing a theoretical foundation for future investigations.

## 6 Conclusion

Digital literacy shows a direct statistical association with older adults’ cognitive function and shows indirect associations with self-efficacy, social participation, and psychological resilience, with self-efficacy showing the strongest mediating effect. These findings support the Conservation of Resources theory, suggesting that digital literacy may be associated with cognitive function through the mobilization of psychological and social resources. Based on these findings, cognitive health interventions for older adults should prioritize digital literacy training to support self-efficacy, social participation, and psychological resilience, which may be associated with the preservation or enhancement of cognitive function.

## Data Availability

The raw data supporting the conclusions of this article will be made available by the authors, without undue reservation.
